# The experiences of patients ill with COVID-19-like symptoms and the role of testing for SARS-CoV-2 in supporting them: A qualitative study in eight European countries during the first wave of the pandemic

**DOI:** 10.1080/13814788.2023.2212904

**Published:** 2023-05-30

**Authors:** Melanie E. Hoste, Marta Wanat, Nina Gobat, Marilena Anastasaki, Femke Böhmer, Sławomir Chlabicz, Annelies Colliers, Karen Farrell, Maria-Nefeli Karkana, John Kinsman, Christos Lionis, Ludmila Marcinowicz, Katrin Reinhardt, Ingmarie Skoglund, Pär-Daniel Sundvall, Akke Vellinga, Herman Goossens, Christopher C. Butler, Alike van der Velden, Sarah Tonkin-Crine, Sibyl Anthierens

**Affiliations:** a Department of Family Medicine and Population Health, University of Antwerp, Antwerp, Belgium; b Laboratory of Medical Microbiology, Vaccine & Infectious Disease Institute, University of Antwerp, Antwerp, Belgium; c Nuffield Department of Primary Care Health Sciences, University of Oxford, Oxford, UK; d Faculty of Medicine, Clinic of Social and Family Medicine, University of Crete, Crete, Greece; e Institute of General Practice, Rostock University Medical Center, Rostock, Germany; f Department of Family Medicine, Medical University of Białystok, Białystok, Poland; g School of Medicine, National University of Ireland, Galway, Ireland; h European Centre for Disease Prevention and Control (ECDC), Solna, Sweden; i Department of Obstetrics, Gynaecology and Maternity Care, Medical University of Białystok, Białystok, Poland; j General Practice/Family Medicine, Primary Health Care, School of Public Health and Community Medicine, Institute of Medicine, Sahlgrenska Academy, University of Gothenburg, Gothenburg, Sweden; k Research, Development, Education and Innovation, Primary Health Care, Region Västra Götaland, Sweden; l School of Public Health, Physiotherapy and Sports Science, University College Dublin, Dublin, Ireland; m Laboratory of Clinical Microbiology, Antwerp University Hospital, Edegem, Belgium; n NIHR Health Protection Research Unit in Healthcare Associated Infections and Antimicrobial Resistance, University of Oxford in Partnership with Public Health England, Oxford, UK; o Julius Center for Health Sciences and Primary Care, University Medical Center Utrecht, Utrecht, The Netherlands

**Keywords:** Qualitative research, SARS-CoV-2, patients, primary care, testing

## Abstract

**Background:**

Access to testing during the first wave of the COVID-19 pandemic was limited, impacting patients with COVID-19-like symptoms. Current qualitative studies have been limited to one country or were conducted outside Europe.

**Objectives:**

To explore - in eight European countries - the experiences of patients consulting in primary care with COVID-19-like symptoms during the first wave of the pandemic.

**Methods:**

Sixty-six semi-structured interviews, informed by a topic guide, were conducted by telephone or in person between April and July 2020. Patients with COVID-19-like symptoms were purposively recruited in primary care sites in eight countries and sampled based on age, gender, and symptom presentation. Deductive and inductive thematic analysis techniques were used to develop a framework representing data across settings. Data adequacy was attained by collecting rich data.

**Results:**

Seven themes were identified, which described the experiences of patients consulting. Two themes are reported in this manuscript describing the role of COVID-19 testing in this experience. Patients described significant distress due to their symptoms, especially those at higher risk of complications from COVID-19, and those with severe symptoms. Patients wanted access to testing to identify the cause of their illness and minimise the burden of managing uncertainty. Some patients testing positive for COVID-19 assumed they would be immune from future infection.

**Conclusion:**

Patients experiencing novel and severe symptoms, particularly those with comorbidities, experienced a significant emotional and psychological burden due to concerns about COVID-19. Testing provided reassurance over health status and helped patients identify which guidance to follow. Testing positive for SARS-CoV-2 led to some patients thinking they were immune from future infection, thus influencing subsequent behaviour.

KEY MESSAGES

- Patients experiencing severe symptoms and/or who are in at-risk groups can experience considerable emotional and psychological stress.
- Patients sought testing to identify if symptoms were caused by COVID-19 and to follow preventive measures to prevent transmission if necessary.
- Testing positive for SARS-CoV-2 was interpreted as having future immunity by some.

## Introduction

In March 2020, Europe became one of the main epicentres for the severe acute respiratory syndrome coronavirus 2 (SARS-CoV-2) pandemic, with strict rules to reduce transmission implemented in many European countries as a means of curbing infection rates and alleviating pressure on healthcare systems during the first wave of the pandemic [1]. To help identify and control transmission of SARS-CoV-2, diagnostic testing is crucial, however; polymerase chain reaction (PCR) testing capacity across Europe was limited and fragmented during the first wave of the pandemic [2]. The World Health Organisation recommended that countries expand their testing capacities to identify and isolate cases to mitigate the spread of the virus [3]. On the other hand, due to the role of person-to-person transmission of SARS-CoV-2 and the importance of adhering to preventive measures, comprehending the significance that patients with COVID-19-like symptoms associate with testing can illuminate areas that can help inform future policies on testing during outbreaks.

Qualitative studies to date explore the experiences of patients with COVID-19 and indicate a collective experience of anxiety related to the novelty of symptoms and the disease and the uncertainty surrounding the trajectory of illness [4–7]. Other studies, investigating the impact of testing, illustrate a link between patient emotional wellbeing and testing, as testing provides reassurance over health status and directs patients towards preventive measures against transmission, influencing their behaviour [8–11]. However, these studies have been conducted with patients who have tested positive for SARS-CoV-2 or were limited to one country.

This manuscript reports part of the results of a qualitative study that aimed to explore the views and experiences of patients with respiratory traction infection (RTI) symptoms presenting to primary care in eight European countries during the COVID-19 pandemic. Here, we report patients’ experiences of being ill with COVID-19-like symptoms and the role of COVID-19 testing in supporting patients. Reporting on these experiences can inform future public health policies in a health emergency.

## Methods

### Study design and patient recruitment

This study is part of a larger project that had two parts: (1) Exploring healthcare professionals’ views and experiences of delivering care to patients with RTI symptoms during the COVID-19 pandemic; and (2) Exploring the views and experiences of patients who consult European primary care services for RTI symptoms during the COVID-19 pandemic. For the patient study, participants were recruited from primary care settings in eight European countries during the first wave of the COVID-19 pandemic between April 2020 and July 2020. Eight countries were purposively selected to get variation in the number of confirmed cases of COVID-19 (assessed in March 2020), health system organisation (taking into account main differences pre-pandemic in relation to organisation of primary care), and geographical location in Europe. The countries included England, Belgium, the Netherlands, Ireland, Sweden, Poland, Greece, and Germany. Countries and research teams were selected from an existing primary care network [12]. A network coordinator from each country had access to primary care sites where patients were recruited.

Patients consulting face-to-face or remotely for COVID-19-like symptoms at primary care sites were eligible for inclusion and were invited to participate in an interview. Parents were invited when patients were aged under 16 years. The local researchers purposively selected patients from those who consented to be contacted for an interview based on age, gender, and symptom presentation. The aim was to interview 8–10 patients per country.

### Interviews

Nine experienced primary care researchers from the primary care networks, with training in qualitative methods, conducted interviews in each country. A standardised semi-structured topic guide was used by all interviewers (Supplementary Material 1). The research team developed this topic guide and asked about experience of symptoms, help-seeking behaviour, the primary care consultation, and COVID-19-related behaviours. Interviews took place by telephone or in person. Patients gave either verbal or written informed consent to take part. Interviews were audio recorded, transcribed verbatim, and translated into English where necessary.

### Data analysis

Analysis was led by MEH using both deductive and inductive thematic analysis techniques [13]. Thematic analysis is a method of identifying patterns and developing themes within data to interpret different aspects of a research topic [13]. Transcripts from Belgium, the Netherlands, England, and Sweden were coded line-by-line, as these were available first, and deductively coded into an a priori framework based on the topic guide and agreed by the core research team (MEH, MW, STC, SA). Data was then coded inductively to create sub-categories before being arranged into themes and sub-themes. This thematic framework was then used to analyse data from the remaining countries. The framework was adapted iteratively to reflect the data in the other countries [13]. Through rich data collection, data adequacy was achieved [14,15]. To ensure rigour, data was discussed within the study team at each stage of the process and within the multidisciplinary study team and interviewers, ongoing analysis was discussed. This was carried out for each country monthly to understand local contexts and to interpret findings. Field notes were kept during data collection that supported these discussions. NVivo 12 was used to support the data analysis.

Due to the breadth of data captured within each analytic theme, the research team decided to report groups of themes in separate manuscripts rather than all themes in a single paper. Some themes have been reported in a prior publication and themes not yet reported and not covered here will be included in a third publication [16]. All themes have been reported in a single report to the funder.

This study was reviewed and received ethical approval in the UK from the sub-committee of the South Central-Berkshire Research Ethics Committee (Reference Number: 20/SC/0175). The seven research networks outside the UK also obtained ethical approval or waivers from their local ethical review committees.

## Results

Sixty-six interviews were conducted between 6 April 2020 and 29 July 2020, each taking 14-55 min (mean 29). Patient characteristics are shown in Table 1. Four patients were children (two in Belgium, one in the Netherlands, and one in Germany) and for each child, a parent was interviewed on their behalf. Almost half of the patients were tested for SARS-CoV-2, however, the proportion of patients tested within a country varied. Table 2 provides contextual information on testing capacity and eligibility during interviews for the different countries. Some patients recruited from the Netherlands had received a test by participating in another research project, however, results were not available at the time of the interviews. Table 3 displays the timing of the interviews in relation to the countries’ lockdowns.

**Table 1.** Patient characteristics.

Country	Number of patients	Age range	Female	Tested for COVID-19	Tested positive for COVID-19	Number of patients in at-risk groups and/or with relevant comorbidities (e.g. age, underlying respiratory illness)	Number of patients who reported experiencing severe symptoms
Belgium	10	7–65	5	1	1	1	3
Ireland	5	32–76	3	3^§^	1	2	2
The Netherlands	10	9–75	3	8^†^	2	3	3
England	12	34–69	7	3	2	5	5
Greece	6	25–63	4	1	0	1	2
Poland	5	26–76	2	3	1	1	2
Sweden	10	19–68	4	7	4	5	5
Germany	8	6–69	6	6	0	0	0
Total	66	6–76	34	32	11	18	22

^§^One patient out of the three who were tested had not received their test result at the time of their interview.

**^†^**Six patients out of the eight who were tested had not received their test results at the time of their interview.

**Table 2.** SARS-CoV-2 testing criteria and capacity of countries during time of interviews.

Country	Testing eligibility criteria	Testing capacity
Belgium [17]	Health professionals with a suspected infection Patients who require hospitalisation	Limited testing capacity
Ireland [18]	Patients with: Two symptoms Have a respiratory illness Were in contact with a confirmed/suspected case In a priority group	Limited testing capacity with long waits for results
The Netherlands [19]	All healthcare workers Patients with severe COVID-19 symptoms who travelled to areas with increased infection risk and/or came in contact with a confirmed case	Limited testing capacity
England [20]	All healthcare workers Patients over the age of 65 with symptoms Patients with severe cases	Strict eligibility criteria for testing and limited testing
Greece [21]	Patients presenting with COVID-19 symptoms such as fever, cough, difficulty breathing	Limited testing capacity
Poland [22]	Patients presenting symptoms	Limited testing capacity with delays in results
Sweden [23]	Patients presenting COVID-19 symptoms	Limited testing capacity
Germany [24]	Testing available for general public	Quick to scale-up testing capacity

**Table 3.** Timing of interviews in relation to national lockdowns*.

Country	Dates of strict lockdown (2020)	Dates of interviews (2020)
Belgium	13 March − 4 May	6 April − 22 April
Ireland	12 March − 18 May	15 April − 13 May
The Netherlands	9 March − 11 May	28 April − 26 May
England	23 March − 10 May	2 May − 8 June
Greece	16 March − 4 May	15 May − 8 July
Poland	12 March − 3 May	11 June − 2 July
Sweden	No strict lockdown	16 June − 29 July
Germany	16 March − 20 April	24 June − 3 July

*National lockdown refers to the closure of schools and non-essential businesses, and travel restrictions.

Seven themes were identified across the interviews that described the experiences of patients consulting in primary care: (i) experiences of being ill during the first wave of the pandemic; (ii) significance of testing for SARS-CoV-2; (iii) seeking healthcare services; (iv) impact of the pandemic on daily life; (v) strategies for prevention of SARS-CoV-2 transmission; (vi) perceptions of media reporting of COVID-19 and vii) opinions on participating in scientific research. This manuscript focuses on two interconnected themes: ‘experiences of being ill during the first wave of the pandemic’ and the ‘significance of testing for SARS-CoV-2′. These themes are discussed here as they were essential to the participants. Reporting on findings in this manner allows us to explore these themes in further detail [25]. Other themes focussed on patients seeking healthcare services have been reported elsewhere [16]. Additional quotes to support the two themes can be found in Supplementary Materials 2 and 3

### Theme 1: The experience of being ill during the first wave

Most patients of the countries in the study, except Germany, described negative emotions associated with their RTI symptoms beyond that of a ‘usual’ RTI. Four factors appeared to exacerbate the emotional and psychological impact of symptoms: the novelty and severity of their symptoms; pre-existing comorbidities and being at risk of developing complications from COVID-19; underlying mental health issues or new mental health issues elicited by their experience; and concern over transmitting the virus to others.

#### Novelty and severity of symptoms

Patients from all countries except Ireland, Poland, and Germany appeared to worry most about symptoms of a new disease, which they had not experienced before. They reported uncertainty about COVID-19, with its fluctuating and potentially vast spectrum of symptoms. Patients, across all countries in the study except Germany, who experienced more severe symptoms were naturally more concerned (Supplementary Material 2). In contrast, patients from Germany did not report any feelings of concern related to their symptoms due to the mildness of their symptoms.

*It’s certainly very worrying; you sleep badly at night and the slightest changes… if you have palpitations or something, you think, ‘Is this the start of something now then?’, going to bed in the evening, and waking up in the middle of the night in a complete panic. There’s a big psychological aspect to this disease as well because nobody knows how it will progress, what will happen and for whom it will happen.’ (P5, 53 years, male – confirmed COVID-19 and in at-risk group – Sweden)*

#### Pre-existing comorbidities and belonging to an at-risk group for COVID-19

In England, Belgium, the Netherlands, Ireland, and Sweden, patients with comorbidities and at risk of developing severe COVID-19 symptoms reported that coping with suspected or confirmed COVID-19 bore a heavy burden. Some patients (from England, Belgium, the Netherlands, and Sweden) believed they would die due to their symptoms, which led to feelings of terror, anxiety, and depression (Supplementary Material 2). Some described ‘putting their affairs in order’ because of this belief.

*It’s probably how I became, how depressed… and my mortal fear. That’s doubtless what’s affected [me the] most – that I’ve talked a great deal about death… And I’ve never felt anything like that before. (P3, 50 years, female – confirmed COVID-19 and in at-risk group – Sweden)*

#### Underlying mental health issues and triggering potential mental health issues

Several patients explicitly expressed feeling particularly low and anxious caused by feeling unwell. For some (in England, the Netherlands, Sweden and Poland), their COVID-19-like symptoms triggered mental health issues they might not have had before being infected. Patients in England who suffered from both physical comorbidities and mental health issues prior to the pandemic reported a considerable emotional burden elicited by their symptoms.

*The whole situation proved to be a great strain on my psyche, I found it hard to recover, I developed an anxiety disorder. I had to take another sick leave to rebuild my mental state. (P2, 28 years, female – suspected COVID-19 – Poland)*

#### Concerns over transmitting the virus to others

In addition, patients from all countries were concerned about potentially transmitting the virus to people who were in at-risk groups and/or other family members. This concern was present for all those with COVID-19-like symptoms, whether confirmed or not (Supplementary Material 2). This prompted patients to take appropriate measures to reduce transmission.

*I wasn’t apprehensive for myself but for other people just those around me. I have many people around me, so in my family sphere, very many who would belong to the risk group. I also have work colleagues who would be in the risk group. I mean I did in fact have concerns there, oh god, what if I have it now and what if I infected someone who maybe won’t get over it as well as I myself? (P7, 34 years, female – negative for COVID-19 – Germany)*

### Theme 2: Significance of testing for SARS-CoV-2

#### Provides confirmation of COVID-19

Most patients in England, Belgium, and Greece had not been tested for SARS-CoV-2 and these patients, in particular, expressed a desire to be tested to confirm whether or not they had COVID-19 (Supplementary Material 3). They believed their symptoms were enough to warrant a test as they felt considerably ill. For these patients, testing meant distinguishing COVID-19 from other comorbidities. However, patients from all countries stressed the importance of being tested to understand what was causing their symptoms.

*I only wished he could’ve tested [me], so I knew. I need to know the difference from my post-polio. But he told me [testing] wasn’t possible… And it’s really a pity that I can’t get tested because it’s worse, how I am now is worse than my post-polio. (P9, 65 years – male – not suspected of COVID-19 and in at-risk group – Belgium)*

#### The perception that testing leads to treatment options

Because testing was seen to offer answers, some patients from England, the Netherlands, and Poland were eager to have access to testing, highlighting that they could not be treated effectively without a diagnosis.

*I think, he [the doctor] should send me for some kind of test. To confirm the disease or rule it out unambiguously. The doctor would then know if the patient was infected or not. How can you order treatment if you don’t know the patient’s disease? (P1, 76 years, male – suspected COVID-19 and in at-risk group – Poland)*

A few patients from the Netherlands and Ireland explained that whilst they were frustrated by the lack of testing, they understood the situation in their country. Others (in England, Germany, and Greece) explained that they felt reassured simply by testing negative and COVID-19 did not cause their symptoms, and as a result, being able to move about freely without worrying about infecting others.

#### Knowing which guidelines to follow if tested positive

A test result meant that some patients (from England, the Netherlands, Ireland, Sweden, and Greece) also knew which preventive measures to follow. Patients in Greece noted that not being recommended for testing by a healthcare professional implied that they did not have to worry about their symptoms. In Ireland, patients stated that although they were relieved that they had been tested, they were frustrated by the delays in acquiring test results as, for some, it took up to two weeks (Supplementary Material 3). Consequently, those who had tested negative had been in self-isolation for two weeks unnecessarily. Notably patients in Germany reported less concern about their symptoms, having had good access to testing and quick results confirming that they did not have COVID-19, easing the burden of symptom uncertainty.

*It was terrific to know that I have it, er… to… well, just to be able to relax, and especially since then now I’m healthy…, even though I’m also obviously still careful, I’m not as afraid as I was before. And it was also good because then I know, OK, but then there are these guidelines I must follow because it’s quite clear what you must do when you have COVID-19. I thought it was good that there were quite clear instructions. (P8, 19 years, female – confirmed COVID-19 – Sweden)*

*Well, since the doctor did not insist on doing it [test], I tried not to worry too much! (P3, 61 years, female – not suspected COVID-19 – Greece)*

#### Belief in immunity if tested positive

Interestingly, in most countries, immunity was often discussed in relation to testing, with some believing that testing positive for SARS-CoV-2 would indicate that they were immune to SARS-CoV-2 (Supplementary Material 3). They expressed a sense of security and relief from having tested positive as they believed it meant immunity to the virus and thus were less anxious about catching it again. Others felt they could visit loved ones or return to work if they ‘knew they were immune’ as there would be little risk of transmitting the virus.

*I'm glad I've had it. When I see the people around me, who haven’t had it, they are quite anxious that they will get infected and in which way. I think I can only be glad that we came out of it this way and might be immune. (P4, 69 years, female – suspected COVID-19 and in at-risk group – The Netherlands)*

## Discussion

### Main findings

As a multi-country study on patients’ experiences managing COVID-19-like symptoms during the first wave of the pandemic, this study illustrates what some patients experienced when little was known about an emergent pathogen.

Our findings demonstrate the emotional and psychological burden experienced by patients with COVID-like symptoms due to the novelty and severity of symptoms they experienced, especially those with pre-existing comorbidities. For some, their ill experiences triggered mental health issues or exacerbated underlying ones whilst others were very concerned about transmitting the virus to others. For patients, testing positive for SARS-CoV-2 confirmed of COVID-19 and was perceived to open up the possibility of treatment options. Testing positive also meant that patients knew which guidelines to follow. Patients had particular expectations of testing, during a period when access to testing was limited in many European countries and some held beliefs about immunity to SARS-CoV-2, when little was known about the disease.

### Strengths and limitations

This unique dataset provides valuable insights at the very early stage of the pandemic. Reporting findings from the first wave is essential as it teaches how to respond to initial uncertainties in a health emergency context. Despite a large number of interviews overall, the number of interviews in each country was relatively small and may not represent the complete picture of the situation in each country. However, the data we collected was rich and allowed us to examine patient’s experiences of testing [26,27]. Extensive discussions with teams in each country aimed to facilitate understanding of the broader issues facing patients at that time and provides us with further contextual data.

Interviews took place during the first wave of the pandemic but at different time points, which could impact patients’ views of testing. Views and behaviour change over time and are influenced by the epidemic curve and national public health measures. We have reported the timing of interviews to help interpret results. The initial data analysis concentrated on data from England, Belgium, the Netherlands, and Sweden, developing a framework that was then applied to the remaining countries. This may have led us to overlook nuances in the data from the latter contexts, however, we adjusted themes and sub-themes as later datasets were analysed and discussed this within the team. An alternative approach would have been to analyse data from different countries separately initially and then to combine them in a common framework; this would have led to delays in our study due to translation issues.

### Comparison with existing literature

A qualitative study in China during the first wave of the pandemic in 2020 exhibited comparable results to this study, with patients describing feelings of uncertainty and anxiety related to their symptoms due to how little was known at the time on the trajectory of COVID-19 [7]. Other more recent qualitative studies on the lived experiences of patients with COVID-19 in different countries reveal that the diversity of symptoms and its unpredictability made patients anxious and fearful [4,6]. In addition, patients with comorbidities reported feeling particularly worried, supporting our findings. Our study strongly reflects the sentiments shared by confirmed COVID-19 patients in other studies, although these studies took place at different time points in the pandemic and in different countries. Negative feelings elicited by both the pandemic and the disease are common and are natural emotions brought on by highly unusual and challenging experiences. Furthermore, most patients in this study have not reported speaking to their primary care clinicians about managing negative feelings brought about by their experiences with being ill with COVID-19-like symptoms. In a different paper where other themes from this study were reported, patients consulted their clinicians for advice and reassurance concerning their symptoms [16]. It illustrates the importance of the role of primary care in the community during public health emergencies as a trusted source of information.

We conducted this study during the first wave of the pandemic when access to PCR testing was limited, no rapid testing was available in many European countries, and when little was known about the consequences of contracting COVID-19. This study recruited a combination of tested and untested patients. Studies on testing in 2020 illustrate links between easing anxiety over health status and protecting loved ones and at-risk populations [5,11]. Our results align with these studies from a similar time frame, indicating that anxiety over health status, especially those with comorbidities, and worries over the risk of transmission to at-risk groups, particularly, influence test-seeking behaviour.

Our study also highlights that some patients whose clinician did not refer them for SARS-CoV-2 testing, mistakenly interpreted this as meaning their clinician was not worried about their illness, which influenced potential transmission behaviours. Whilst our study did not fully explore patients’ perceptions on the accuracy of test results, those who tested negative assumed that they were no longer at risk and did not further question the validity of their tests. Studies on rapid antigen testing amongst students in the UK and teachers in Belgium illustrate that the results of their test somewhat determined their behaviour, and by testing negative, they were less likely to adhere to preventive measures [10,28].

Lastly, studies at different and later periods of the pandemic showed that testing uptake was low, with some increase only during outbreak periods due to a lower perceived risk of contracting and/or developing severe COVID-19 symptoms [26,29].

In addition, our data shows considerable patient uncertainty behind the implications of having had an infection with SARS-CoV-2 and immunity, which directly reflects scientific evidence at the time. To date, there is no existing qualitative literature on patients’ beliefs about immunity at this time during the pandemic. Patients made assumptions as to what testing positive for SARS-CoV-2 would mean, informing their later behaviour.

### Implications of findings

With primary care optimally placed as trusted points of contact, we propose that professionals in these settings be trained and supported to provide care for those who need emotional and psychological support in dealing with novel and severe symptoms. Future policies could consider primary care as a setting where patients can be sign-posted to sources for support during times of a public health emergency.

Our results suggest that testing may reduce the burden of uncertainty regarding symptoms experienced and could direct patients on their next course of action. Therefore, it is important to consider the benefits of providing easy access to testing for patients and rapid test results, even in the initial stages of the pandemic. In conjunction, communication around the validity of results is crucial to avoid assumptions and information on the importance of maintaining preventive measures can still be prudent for patients who receive a negative outcome. Additionally, patients may assume immunity after being confirmed with COVID-19, which can lead to non-adherent behaviour against preventative measures. It is our view that this needs to be taken into consideration when communicating these messages to patients to avoid misunderstandings.

In our view, countries’ public health campaigns should appeal to the public’s desire to protect family and friends if they want to encourage testing. We base this on our study, alongside others [9,27], that demonstrated that patients had significant concerns about infecting loved ones which motivated patients to seek healthcare advice and attend testing.

## Conclusion

Managing symptoms of a new infection can carry a significant burden for patients depending on the novelty of symptoms, the severity, and any comorbidities patients may have. Additionally, patients were concerned about the transmission of the virus to loved ones. Patients sought testing for SARS-CoV-2 as they believed it would provide a diagnosis and access to treatment. A positive test result enabled patients to identify which guidelines they should follow. Lastly, after testing positive, patients made assumptions about immunity, which influenced their subsequent behaviour.

## Supplementary Material

Supplementary Material 3

Supplementary Material 2

Supplementary Material 1

## Data Availability

The datasets generated during the current study are not publicly available as the research programme is ongoing but are available from the corresponding author upon reasonable request.
