# Supplementary material for: The experiences of patients ill with COVID-19-like symptoms and the role of testing for SARS-CoV-2 in supporting them: A qualitative study in eight European countries during the first wave of the pandemic
Source: Eur J Gen Pract. 2023 May 30;29(2):2212904. doi: 10.1080/13814788.2023.2212904 (PMC10249444; doi:10.1080/13814788.2023.2212904)
Supplement: Supplementary Material 3 [file IGEN_A_2212904_SM1341.docx]

**Supplementary Material 3. Supporting quotes for Theme 2 ‘Significance of testing for SARS-CoV-2’**

| **Provides confirmation of COVID-19** | *‘It [testing] was done later in the evening in the Emergency Clinic with this swab via your nose and throat. Quite uncomfortable but it was welcome because I was then able to get an answer to ‘What’s happening?’ So, you had to wait until the following day to get it; you got it on the round.’* (P1, 53 years, male – confirmed COVID-19 – Sweden)  *‘Okay, I was relieved, in the end there was no chance that I had passed something on to anyone. I did not have it, I did not transmit it. It was a relief. Before I knew the result, I was concerned because while I was waiting outside before the test, people passed by, acquaintances, and wanted to talk to me and greet me and I told them to keep their distance.’* (P4, 56 years, female – negative COVID-19 – Greece) |
| --- | --- |
| **The perception that testing leads to treatment options** | *‘After I saw my GP, I received a text that I was COVID-19 positive. Because of this, I contacted the GP again in X and during that time my cough was worsening. It’s not too bad really; there’s a slight breathlessness on exertion when climbing stairs. So the GP thought because of that – over a week of fever plus coughs and then some breathlessness, they prescribed doxycycline for me. After doxycycline my fever – I felt like it disappeared and I wasn’t out of breath anymore on exertion. Then they prescribed another amoxicillin dose because my fever was still high.’* (P5, 34 years, female – confirmed COVID-19 – England) |
| **Knowing which guidelines to follow if tested positive** | *‘Interviewer: And when did you receive your test result?’*  *P1: I have not received them yet.*  *Interviewer: You still didn’t receive them? How long ago was that?*  *P1: It was a fortnight last Monday. It makes me feel frustrated but on the other hand, I know that there is a huge backlog.’* (P1, 59 years, female – confirmed COVID-19 and in at-risk group – Ireland)  *‘I received the test result at the end of June through my family doctor. He had just called me because he got the fax and it was negative. And afterwards, for me, it was like, sure, okay, good, you can sit back and relax, you don’t need to inform anyone or anything, you can move around normally without having to fear that there is something.’* (P7, 34 years, female – negative COVID-19 – Germany) |
| **Belief in immunity if tested positive** | *‘I know they’re not going to confer lifelong immunity – they won’t know that – but at least... For instance, if I could have one today, and I had antibodies to show that I had it, I could see my grandchildren and my children who live 500 yards away…’* (P9, 62 years, female – suspected COVID-19 and in at-risk group – England)  *‘I think that if there was more clarity in one way if you would be able to test whether you are infected or have been infected… If there could be clarity about the fact that if you were infected, are you immune, that you just have clarity… More clarity, I think. That tests can be carried out, but continuously... I'm immune, although I don't know... Can you still be a carrier or not? Because then you can still infect other people, apart from being... If you could actually know that you are not a danger to others... I think that yes… that that would provide peace of mind.’* (P3, 43 years, female – suspected COVID-19 – Belgium) |
