# Supplementary material for: The experiences of patients ill with COVID-19-like symptoms and the role of testing for SARS-CoV-2 in supporting them: A qualitative study in eight European countries during the first wave of the pandemic
Source: Eur J Gen Pract. 2023 May 30;29(2):2212904. doi: 10.1080/13814788.2023.2212904 (PMC10249444; doi:10.1080/13814788.2023.2212904)
Supplement: Supplementary Material 2 [file IGEN_A_2212904_SM1340.docx]

**Supplementary Material 2. Supporting quotes for Theme 1 ‘The experience of being ill during the COVID-19 pandemic’**

| **Novelty and severity of symptoms** | *‘I have been anxious for a while of something because very little is known about... Um... and yet I don't know yet how worried I should be about any long-term damage to the lungs…’* (P2, 27 years, female – suspected COVID-19 – Belgium)  *‘I had never experienced anything like that before, it was really horrifying. I was trying to get up but I couldn’t. When I managed to use a thermometer and I took my temperature, it was 42°C. I was in this state for almost an hour and sweating all over. After that, I tried to get up again and I did. I was sure it was a coronavirus infection… Now it’s much better but I was panic-stricken when I had those symptoms because I can only count on myself.’* (P1, 76 years, male – suspected COVID-19 and in at-risk group – Poland) |
| --- | --- |
| **Pre-existing comorbidities and belonging to an at-risk group for COVID-19** | *‘It’s been tough. I’m quite a strong person but it scared the living hell out of me, to be honest. I’ve had chest infections before; I had pneumonia last year, and to be honest, it’s the lowest that I’ve ever been and the only time I would ever honestly say I truly believe that I’m going to die, because it... nothing I did was working, and I think it’s just the unknown of it, I think, which is really scary, and the fact that there is no real treatment, per se, for it, it’s a case of you have to ride it kind of out, and I think that’s the scary part.’* (P8, 36 years, female – suspected COVID-19 and in at-risk group – England)  *‘Yeah, that was pretty emotional. In that way, we both said: ‘It could have been so bad that we both wouldn't have been here anymore.’ You get the impact when it's all over. At that moment, you feel what could have happened.’* (P4, 69 years, female – suspected COVID-19 and in the at-risk group – The Netherlands) |
| **Underlying mental health issues and triggering potential mental health issues** | *‘It was quite bad at the time. I got very depressed afterwards. I’d had these awful nights of feverishness, sweating and feeling dreadful, and no appetite or anything… I take Fluoxetine to help with depression, so it’s not unusual but that was quite unusual in that I just sank so low suddenly, and for about three or four days I felt really, really low and then got back to normal again.’* (P9, 62 years, female – suspected COVID-19 and in at-risk group – England)  *‘I think in the moment I was depressed. Mostly because I had no idea when it would end. It just didn’t stop. I became more and more sombre. You just don’t feel well. You feel bad; you’re not able to do as much.’* (P7, 30 years, female – suspected COVID-19 – The Netherlands) |
| **Concerns about transmitting the virus to others** | *‘About 70 people are working for me, I didn’t want to make them sick. So for me that was, I just can’t do that despite how stubborn I am. I can’t transmit it to somebody because in my case, I’ve been sick and I’ve felt miserable but I didn’t need hospital admission. Imagine that I infect somebody and that person ends up in hospital or even worse… yeah you don’t want to have that on your hands.’* (P1, 47 years, female – suspected COVID-19 – The Netherlands)  *‘Yes, there was my husband but because of the situation, I was afraid to have something and infect them… Look, until I fully recovered, I had this insecurity and fear, what I finally had. I could not see my parents while they were downstairs for about a month. So on with our friends and relatives. I felt like I might have something and transmit it to the rest.’* (P5, 45 years, female – suspected COVID-19 – Greece) |
