# Supplementary material for: The experiences of patients ill with COVID-19-like symptoms and the role of testing for SARS-CoV-2 in supporting them: A qualitative study in eight European countries during the first wave of the pandemic
Source: Eur J Gen Pract. 2023 May 30;29(2):2212904. doi: 10.1080/13814788.2023.2212904 (PMC10249444; doi:10.1080/13814788.2023.2212904)
Supplement: Supplementary Material 1 [file IGEN_A_2212904_SM1339.docx]

**Supplementary Material 1. Topic guide for patient interviews**

**Below is a list of topics to be discussed in this study.**

1. **Participants’ beliefs about symptoms of COVID-19.**
2. **Participants’ reports of help-seeking behaviour during the SARS-CoV-2 outbreak.**
3. Participants’ perceptions and experiences of care received.
4. Participants’ perceptions of personal risk of COVID-19 and experiences of prevention behaviours before and after consultation.
5. Participants’ views on public health messages.

**Example questions (additional questions may be added following the topics above):**

**Symptoms and help-seeking**

1. **You recently consulted your health service for respiratory symptoms, please tell me why you consulted and what happened.**
2. **What symptoms did you have and what were you most concerned about?**
3. **Did you try anything to help manage your symptoms at home?**
4. **Where did you go to get advice about your symptoms? How useful did you find this advice? What additional information do you think should be available?**
5. **When did you decide to consult? Why?**

**The consultation**

1. **What did you think of the consultation that you had?**
2. **What went well in your consultation? Is there anything the GP could have said or done that would have helped you more?**
3. **In your consultation, you had a coronavirus test; how did you feel about having this test?**
4. **When did you receive your test result and what happened next?**

**Prevention behaviours**

1. **What are you doing now to prevent yourself from getting coronavirus?**
2. **Have you changed anything you do since your consultation?**

**Public health messages**

1. **What public health messages have you seen about coronavirus? Where have you seen these?**
2. **What do you think about these public health messages?**

**Closing**

1. **Is there anything else you want to discuss about coronavirus or your experiences?**
